# Implementation of rectangular slit-inserted ultra-wideband tapered slot antenna

**DOI:** 10.1186/s40064-016-3033-4

**Published:** 2016-08-22

**Authors:** Sun-Woong Kim, Dong-You Choi

**Affiliations:** 1 Department of Information and Communication Engineering, Graduate School of Chosun University, Gwangju, Korea; 2 Department of Information and Communication Engineering, Chosun University, Gwangju, Korea

**Keywords:** Ultra wideband, Tapered slot antenna, Rectangular slit, Directivity radiation pattern

## Abstract

In this paper, a tapered slot antenna capable of ultra-wideband communication was designed. In the proposed antenna, rectangular slits were inserted to enhance the bandwidth and reduce the area of the antenna. The rectangular slit-inserted tapered slot antenna operated at a bandwidth of 8.45 GHz, and the bandwidth improved upon the basic tapered slot antenna by 4.72 GHz. The radiation pattern of the antenna was suitable for location recognition in a certain direction owing to an appropriate 3 dB beam width. The antenna gain was analyzed within the proposed bandwidth, and the highest gain characteristic at 7.55 dBi was exhibited at a 5-GHz band. The simulation and measurement results of the proposed tapered slot antenna were similar.

## Background

The number of applications for wireless communication systems are increasing because of interest from industry, the medical and scientific communities, and various others. Accordingly, in the academic field, many studies on antennas types such as wideband, multiband, and compact are being conducted.

In this paper, an antenna capable of wideband communication is proposed. Possible antenna structures include bow-tie, log-periodic, spiral, fractal, and tapered slot. The antennas with these structures are applied to various wireless communication systems depending on the antennas’ shape and characteristics, and particularly in the UWB communication system (Kim et al. 2015; Yoon et al. 2015).

For the UWB wireless communication system, the allowed limit was defined as the frequency band of 3.1–10.6 GHz by FCC (Federal Communication Commission) in USA, and it has to satisfy the 25 % of fractional bandwidth and the frequency bandwidth of 500 MHz or higher (Choi et al. 2014).

UWB technology has many applications including penetrating radar, nondestructive testing radars for civil engineering, precision location tracking systems, and medical and wireless communications (Notice of Inquiry in the Matter of Revision of Part 15 of the Commission’s Rules Regarding Ultra-Wideband Transmission Systems; Garg et al. 2001; Deng et al. 1999; McKinney et al. 2008).

The antenna proposed in this paper is a tapered slot antenna. The tapered slot antenna is a directive antenna, which can recognize a location in a certain direction, and has many applications including measurement systems, vehicular radar, and through-wall radar.

In this paper, a tapered slot antenna is designed and proposed. The antenna has a 3 dB beam width, high gain, and directive characteristics, which are appropriate for reducing the area of the antenna and for location recognition. The bandwidth was improved by inserting rectangular slits in the radiator of the antenna.

This paper is organized as follows. In “Materials and methods” section, the tapered slot antenna is designed and proposed. In “Results and discussion” section, the characteristics of antenna are analyzed through simulation and measurement. In “Conclusions” section, a conclusion is provided.

## Methods

The tapered slot antenna can be easily fabricated because of its dimensional tolerance of low precision and infinite bandwidth. Nevertheless, a desired bandwidth can be induced by changing the physical size of the radiator and using various design technologies. The design and simulation analysis of the proposed antenna is obtained by using an HFSS of Ansys Co.

**Fig. 1:**
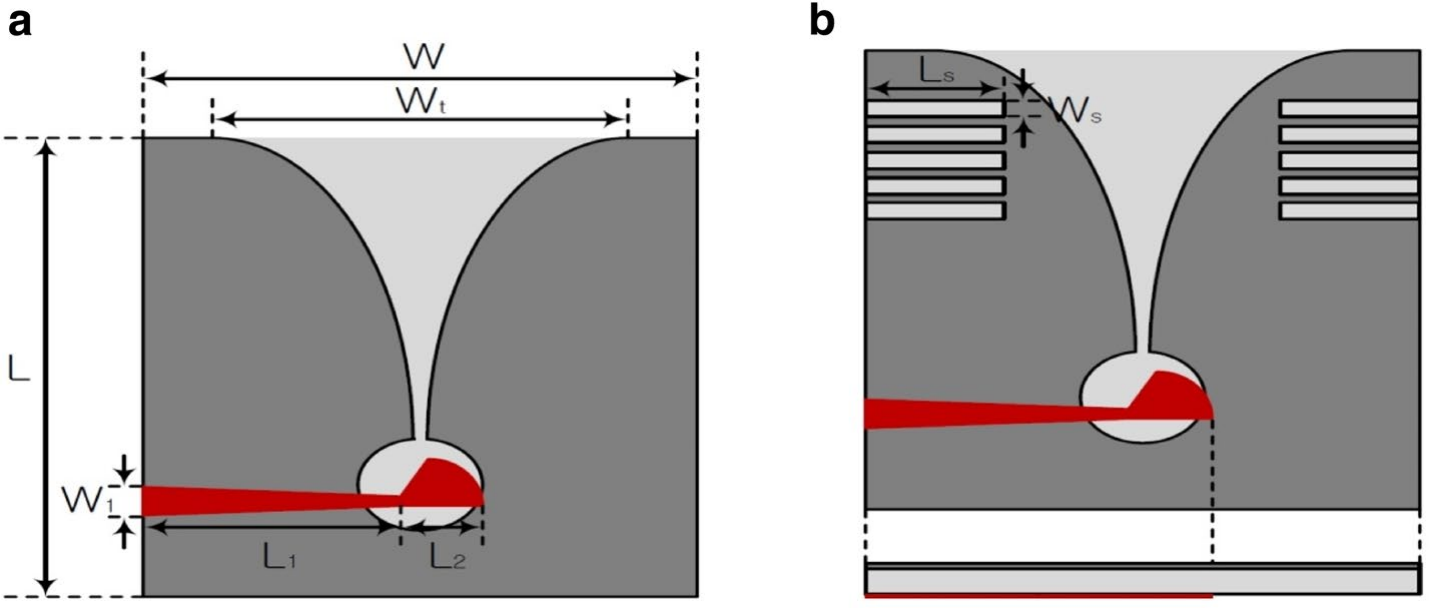
Structure of proposed tapered slot antenna. **a** Structure-1. **b** Structure-2

A wide bandwidth was induced by inserting rectangular slits in Structure-1, as shown in Fig. 1a. The structure of the proposed antenna is shown in Fig. 1b (Fei et al. 2011; He et al. 2014). The proposed antenna was improved wide impedance bandwidth by inserting rectangular slits in the radiator of the antenna, and the area of the antenna is achieved a reduction ratio of 15.5 %. However, the proposed antenna was satisfied with the commercial UWB band.

**Fig. 2:**
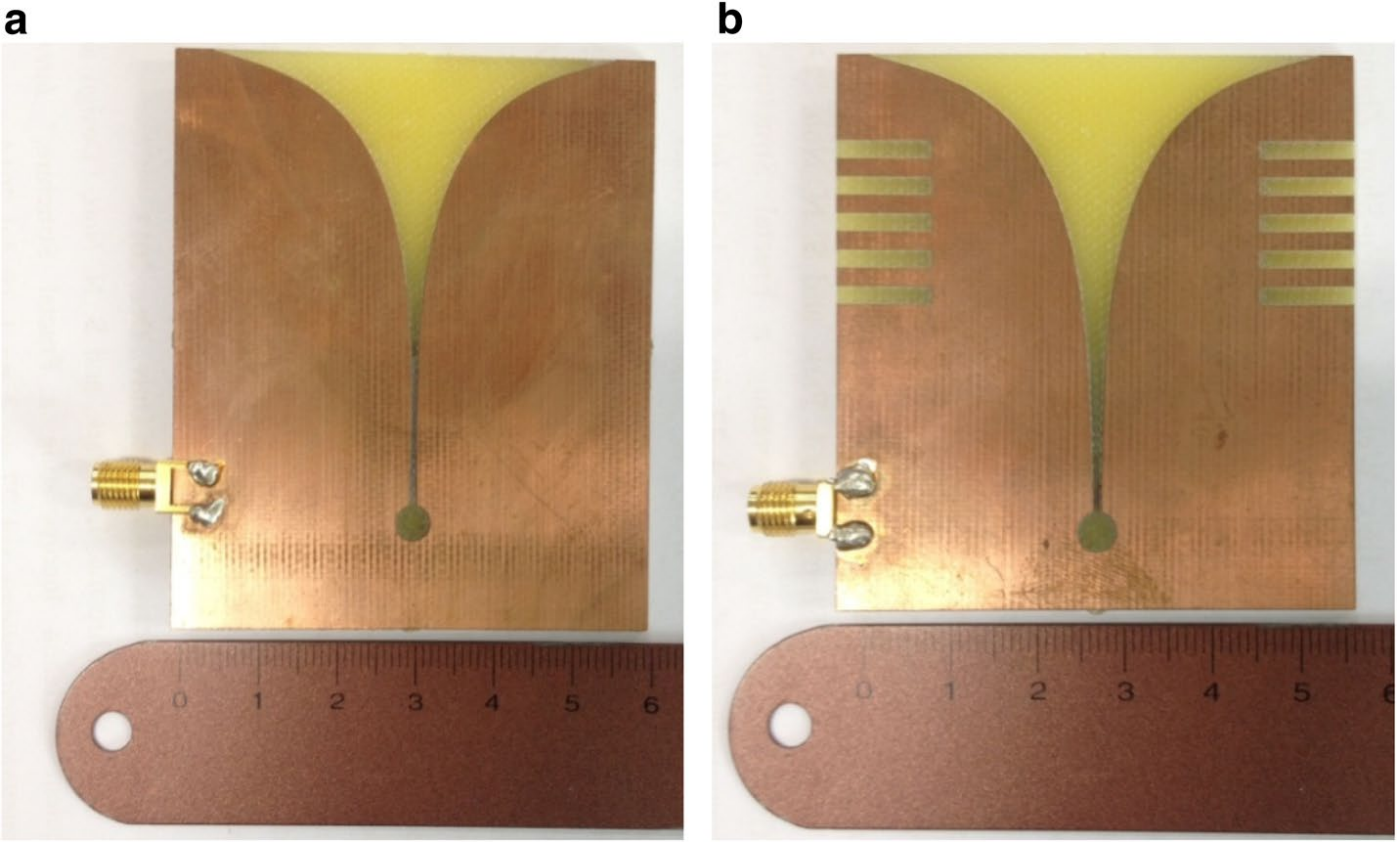
Structure of fabricated antenna. **a** Structure-1. **b** Structure-2

The proposed tapered slot antenna was fabricated on an FR-4 substrate with a specific inductive capacity of 4.7, a loss tangent of 0.019, and a thickness of 1.6 mm (Kim et al. 2015). Once the aperture size of the antenna is determined by operating frequency, the antenna should be able to transmit a signal at its longest wavelength. To do this from a dielectric substrate, Eq. (1) must be satisfied:

1 $$W_{t} = \frac{{\lambda_{g} }}{2} = \frac{C}{{2f_{r} \sqrt {\varepsilon_{r} } }}$$

where *λ*_*g*_ is the guide wavelength (m), *C* is the speed of light (m/s), and *ε*_*r*_ is the dielectric constant. In Eq. (1), the tapered slot antenna operates as a resonance antenna at a low frequency, and is determined by the physical size of the operating frequency *f*_*r*_ and the value of *ε*_*r*_ used in the fabrication of the antenna. Therefore, by using Eq. (1), the total size of the tapered slot antenna can be determined for operation in a desired bandwidth. The fabricated antenna and its dimensions are shown in Fig. 2 and Table 1 Kim (2013).

**Table 1:** Dimensions of proposed antenna (mm)

Antenna	Structure-1	Structure-2
*W*	60	58.2
*L*	70	61
*W*_*t*_	51	52
*W*_1_	3	3
*L*_1_	28	28
*L*_2_	7.5	7.4
*W*_*s*_	–	2
*L*_*s*_	–	10.7

*W* and *L* are the length and width of the proposed antenna, and *W*_*t*_ is the aperture width of the tapered slot. *W*_1_, *L*_1_, and *L*_2_ are the lengths and width of the impedance converter. *W*_*s*_ and *L*_*s*_ are the width and length of the rectangular slit for the proposed tapered slot antenna (Choi et al. 2014; Shrestha et al. 2013).

## Results and discussion

### Analysis of matching characteristics and antenna bandwidth

The reflection coefficient Γ is the amount of signal reflection by impedance mismatch that occurs between the source and antenna during the operation of an antenna in a single-port circuit. The optimal reflection coefficient is Γ = 0, and the usual impedance bandwidth of the antenna is defined as −10 dB S_11_ and VSWR ≤ 2. This means that approximately 11 % of the input power is reflected (Chang 2000).

**Fig. 3:**
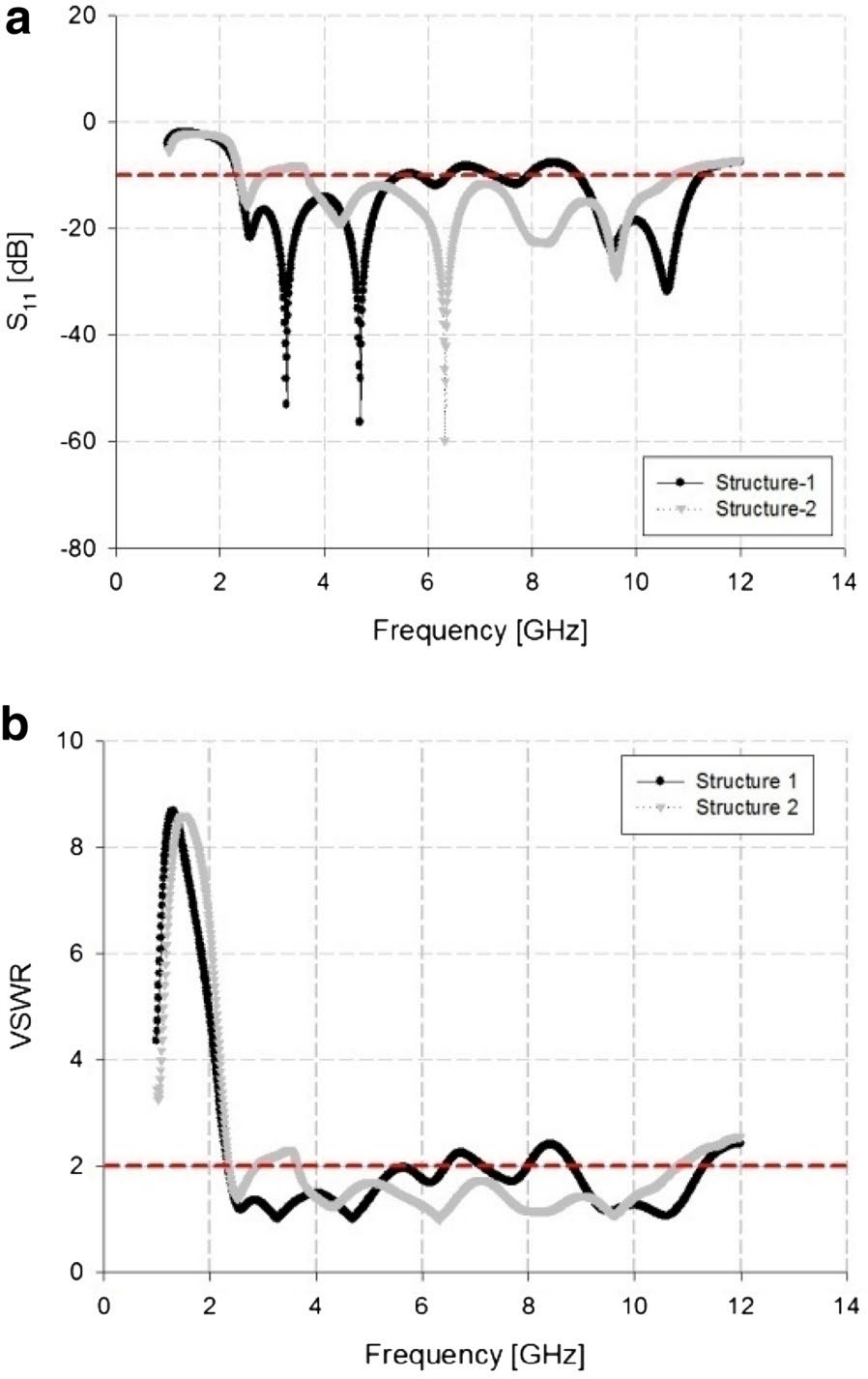
Results of impedance bandwidth simulation. **a** S_11_. **b** VSWR

Figure 3 shows the results of an impedance bandwidth simulation for Structure-1 and Structure-2 of the proposed tapered slot antenna.

In the simulation results shown in Fig. 3, the impedance bandwidth of Structure-1 satisfied the requirements of −10 dB S_11_ and VSWR ≤ 2 in the low-frequency bandwidth of 2.33–6.46 GHz and high frequency-bandwidth of 8.84–11.32 GHz. However, the middle-frequency bandwidth was suppressed. By contrast, in the case of a rectangular slit-inserted tapered slot antenna, the 7.2 GHz bandwidth satisfied the requirements for −10 dB S_11_ and VSWR ≤ 2 in the 3.64–10.84 GHz bandwidth, and improvement was seen in the middle-frequency bandwidth of Structure-1.

**Fig. 4:**
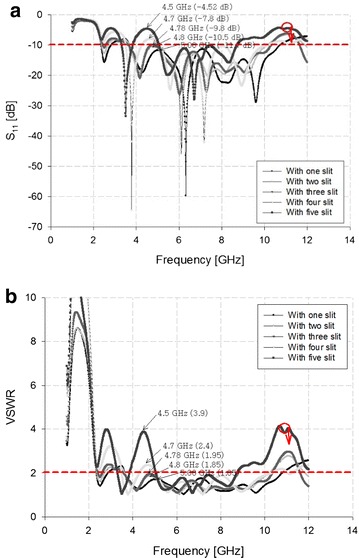
Simulation analysis through the increase of the rectangular slit. **a** S_11_. **b** VSWR

The improved impedance bandwidth was observed through the increase of rectangular slits, as show in Fig. 4.

In the simulation results show in Fig. 4, a steady matching characteristics are obtained due to the insertion of rectangular slits which result in increase of impedance bandwidth.

**Fig. 5:**
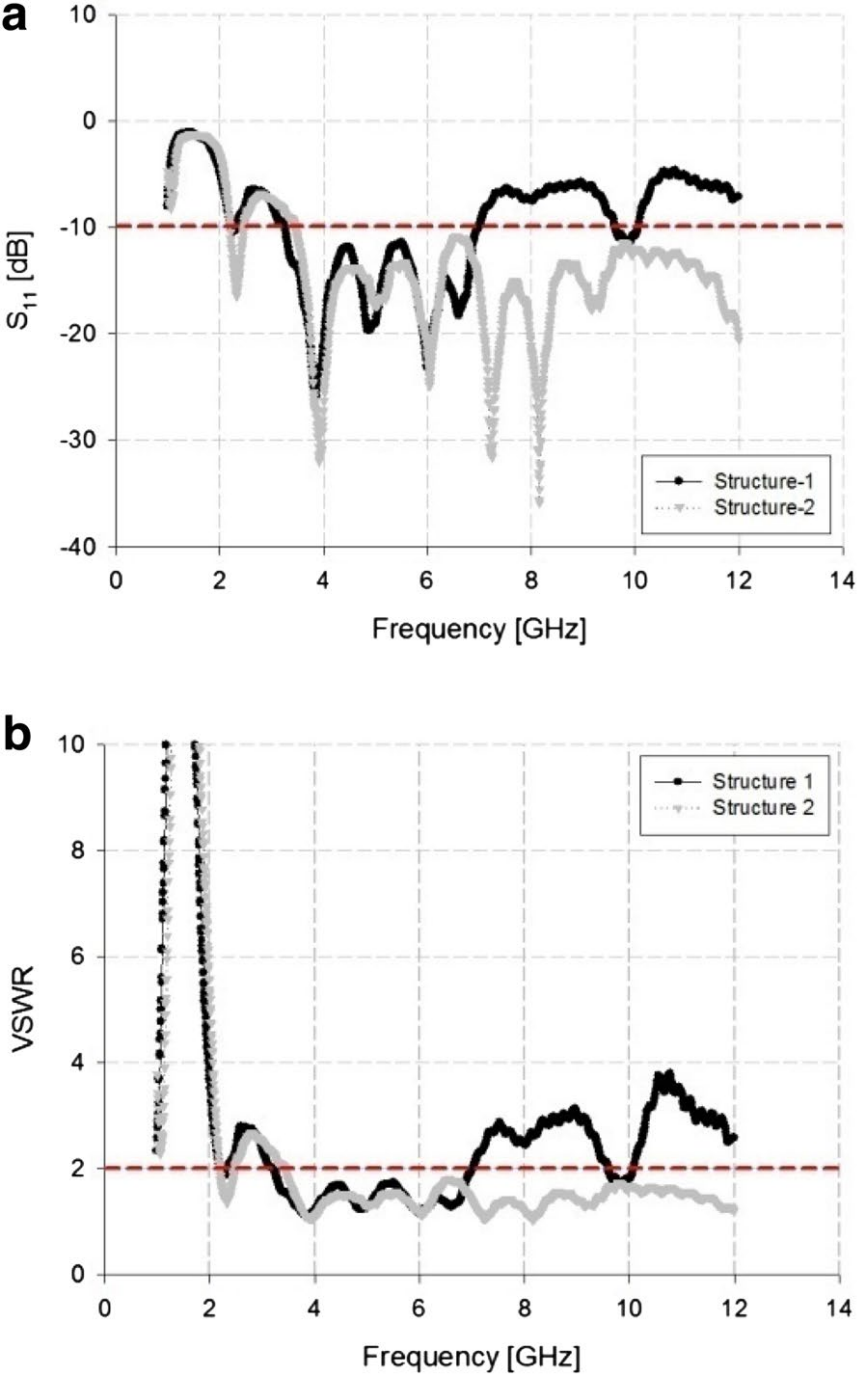
Measurement results for impedance bandwidth. **a** S_11_. **b** VSWR

The impedance bandwidth of the proposed antenna was measured by Network Analyzer (N5230A) of Agilent Co. Pozar (2012). The results of fabricating the tapered slot antenna using the impedance bandwidth measurement results is shown in Fig. 5.

In the measurement results shown in Fig. 5, Structure-1 showed a bandwidth of 3.73 GHz by satisfying −10 dB S_11_ and VSWR ≤ 2 in the 3.26–6.99 GHz bandwidth range. While satisfying −10 dB S_11_ and VSWR ≤ 2 in the 3.55–12 GHz bandwidth similarly, Structure-2 achieved a bandwidth of 8.45 GHz. The simulation and measurement results are similar, and the measurements show that the bandwidth of 4.72 GHz improved in Structure-2 compared with Structure-1.

### Analysis of antenna gain and radiation pattern

**Fig. 6:**
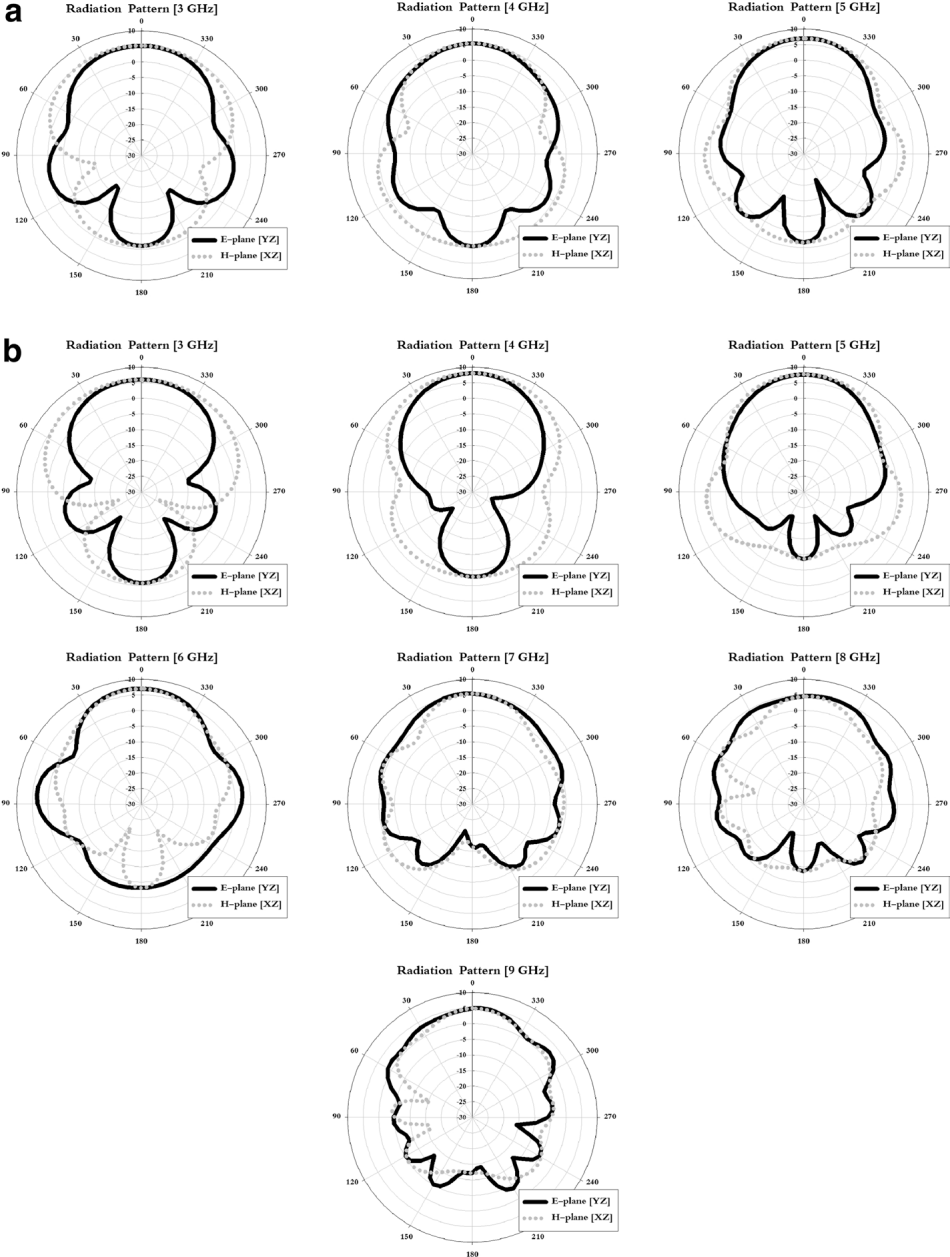
Results of radiation pattern simulation. **a** Structure-1. **b** Structure-2

One important antenna characteristic is how much the antenna can focus and radiate radio waves in a certain direction. In the E-plane (*x*–*z*) and H-plane (*x*–*y*) of the proposed tapered slot antenna, the radiation pattern and 3 dB beam width were analyzed. The analysis of the simulation is shown in Fig. 6.

In the simulation results of Fig. 6 for the E-plane and H-plane, the 3 dB beam width was 130° and 77° in the 3 GHz band. For Structure-1, the 3 dB beam width was 85° and 77° in the 4 GHz band, and 78° and 65° in the 5 GHz band. For Structure-2, the beam width was 140° and 75° in the 3 GHz band, 95° and 110° in the 4 GHz band, 84° and 64° in the 5 GHz band, 62° and 55° in the 6 GHz band, 55° and 68° in the 7 GHz band, 42° and 90° in the 8 GHz band, and 41° and 85° in the 9 GHz band.

**Fig. 7:**
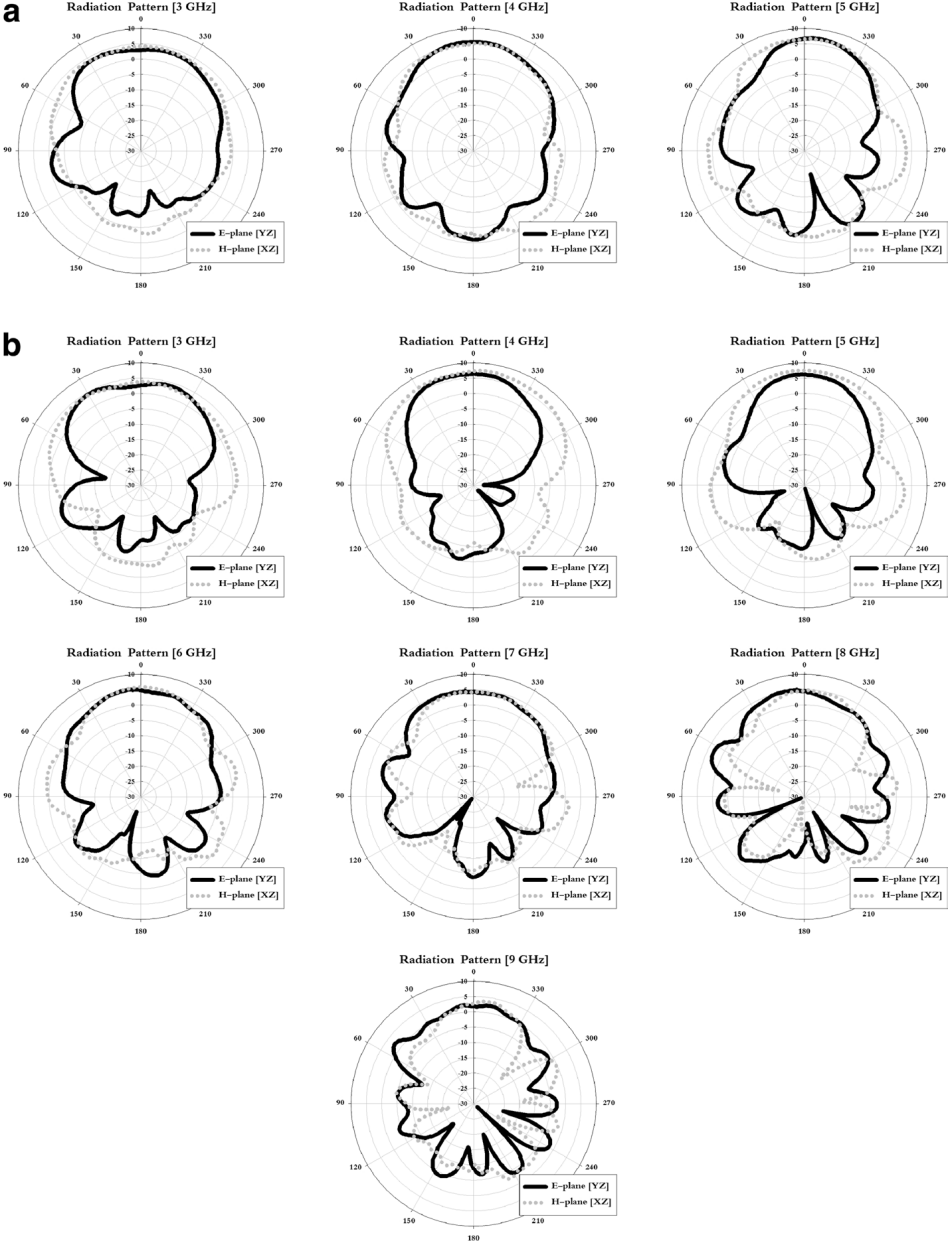
Results of radiation pattern measurement. **a** Structure-1. **b** Structure-2

Radiation pattern measurement of the proposed antenna is measured using an antenna far-field analysis system in an anechoic chamber as mentioned in Chang (2000). The radiation pattern measurement results for the tapered slot antenna that was fabricated using these characteristics are shown in Fig. 7.

Examining the measurement results in Fig. 7, in the E-plane and H-plane, the 3 dB beam width was 90° and 125° in the 3 GHz band for Structure-1, 75° and 80° in the 4 GHz band, and 50° and 75° in the 5 GHz band. For Structure-2, the beam width was 115° and 128° in the 3 GHz band, 60° and 123° in the 4 GHz band, 55° and 84° in the 5 GHz band, 78° and 58° in the 6 GHz band, 87° and 80° in the 7 GHz band, 58° and 55° in the 8 GHz band, and 48° and 42° in the 9 GHz band.

In the simulation and measurement results, Structure-1 and Structure-2 exhibited an approximate 3-dB beam width. In addition, because of the directive radiation pattern, a characteristic suitable for location recognition in a certain direction was seen.

**Fig. 8:**
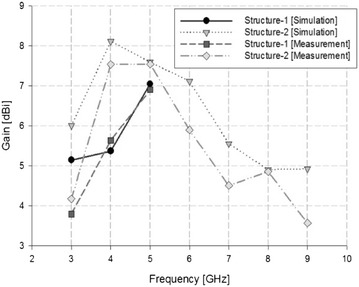
Antenna gain simulation and measurement results

Based on the radiation pattern analysis, the antenna gain in the proposed bandwidth was analyzed. Figure 8 shows the simulation and measurement results for Structure-1 and Structure-2.

In the simulation results for Structure-1, a gain of 5.15 dBi was seen in the 3 GHz band, 5.37 dBi in the 4 GHz band, and 7.05 dBi in the 5 GHz band. In the case of Structure-2, the gain was 6.06 dBi in the 3 GHz band, 8.12 dBi in the 4 GHz band, 7.6 dBi in the 5 GHz band, 7.12 dBi in the 6 GHz band, 5.56 dBi in the 7 GHz band, 4.91 dBi in the 8 GHz band, and 4.93 dBi in the 9 GHz band. In Structure-1, the antenna gain measurement results showed a gain of 3.8 dBi in the 3 GHz band, 5.64 dBi in the 4 GHz band, and 6.91 dBi in the 5 GHz band. In the case of Structure-2, the gain was 4.18 dBi in the 3 GHz band, 7.54 dBi in the 4 GHz band, 7.55 dBi in the 5 GHz band, 5.9 dBi in the 6 GHz band, 4.51 dBi in the 7 GHz band, 4.86 dBi in the 8 GHz band, and 3.57 dBi in the 9 GHz band.

Hence, the proposed antenna’s simulation and measurement results for antenna gain were similar.

### Comprehensive analysis results for antenna

Table 2 shows the comprehensive analysis results for the proposed antenna.

**Table 2:** Comprehensive analysis results

			Simulation	Measured
Bandwidth		Structure-1	4.13 GHz	3.73 GHz
		Structure-2	7.2 GHz	8.45 GHz
−10 dB S_11_ and VSWR ≤ 2		Structure-1	2.33–6.46 GHz	3.26–6.99 GHz
		Structure-2	3.64–10.84 GHz	3.55–12 GHz
Antenna gain				
3 GHz		Structure-1	5.15 dBi	3.8 dBi
4 GHz		Structure-1	5.37 dBi	5.64 dBi
5 GHz		Structure-1	7.05 dBi	6.91 dBi
3 GHz		Structure-2	6.06 dBi	4.18 dBi
4 GHz		Structure-2	8.12 dBi	7.54 dBi
5 GHz		Structure-2	7.6 dBi	7.55 dBi
6 GHz		Structure-2	7.12 dBi	5.9 dBi
7 GHz		Structure-2	5.56 dBi	4.51 dBi
8 GHz		Structure-2	4.91 dBi	4.86 dBi
9 GHz		Structure-2	4.93 dBi	3.57 dBi
Antenna area		Structure-1	4200 mm^2^	
		Structure-2	3550 mm^2^	

In Table 2, the antenna impedance bandwidth was improved in Structure-1 compared with Structure-2, and the simulation and measurement results in simulation antenna gain. Furthermore, the total area of the antenna was 4200 mm^2^ for Structure-1 and 3550 mm^2^ for Structure-2. Structure-2 showed a reduction in area of 15.5 % compared with Structure-1. The impedance bandwidth of the antenna was satisfied −10 dB S11 and VSWR ≤ 2, and it shows good impedance matching characteristics.

However, the simulation and measured analysis of the proposed antenna shows the mismatch. A mismatch is considered in the two kinds. The first pertained to loss during the manufacturing process, and the second is a mismatch between the antenna and connector.

The proposed antenna and different antennas are compared in Table 3. The proposed antenna is compact design and it has the wide bandwidth characteristic. This features of proposed antenna of Structure 2 are more detailed in Figs. 3 and 5 which emphases on simulated and experimental results.

**Table 3:** Comparison of the proposed antenna and different antennas

Antenna	Impedance bandwidth (GHz)	Dimensions (mm^2^)
Shao et al. (2013)	0.64–6	130 × 70
Herzi et al. (2016)	2–5	90 × 120
Wang et al. (2016)	0.65–5.9	220 × 170
Proposed antenna	3.5–12	61 × 58.2

## Conclusions

A directive antenna suitable for use as an ultra-wideband tapered slot antenna and for location recognition was designed and proposed. To improve the bandwidth, rectangular slits were inserted in the tapered slot antenna, and the antenna area was reduced. The fabricated rectangular slit-inserted tapered slot antenna exhibited a bandwidth of 8.45 GHz and improved its bandwidth by approximately 4.72 GHz. Furthermore, the antenna showed a 15.5 % reduction in area.

The antenna radiation pattern exhibited a beam width of 3 dB that was suitable for all bandwidths, and a directivity characteristic that was suitable for location recognition in a certain direction. Its highest antenna gain, which was 7.55 dBi, was seen in the 5 GHz band.

After these characteristics were comprehensively analyzed, the proposed antenna was shown to be suitable for location recognition in a certain direction owing to its impedance bandwidth characteristic of ultra wideband and its radiation pattern that showed directivity.
